# Ca^2+^ and Ca^2+^-interlocked membrane guanylate cyclase signal modulation of neuronal and cardiovascular signal transduction

**DOI:** 10.3389/fnmol.2015.00007

**Published:** 2015-03-06

**Authors:** Rameshwar K. Sharma, Wolfgang Baehr, Clint L. Makino, Teresa Duda

**Affiliations:** ^1^Research Divisions of Biochemistry and Molecular Biology, The Unit of Regulatory and Molecular Biology, Salus UniversityElkins Park, PA, USA; ^2^School of Medicine, Department of Ophthalmology and Visual Sciences, University of UtahSalt Lake City, UT, USA; ^3^Massachusetts Eye and Ear Infirmary and Harvard Medical SchoolBoston, MA, USA

**Keywords:** cyclic GMP, Ca^2+^, membrane guanylate cyclase, multiple transduction modes, signal transduction

The papers in this research topic are focused on the extraordinary tale of cyclic GMP: its recognition as a hormonal second messenger and its subsequent interplay with intracellular calcium where the two together become co-messengers in cellular signal transduction processes. In this emerging theme membrane guanylate cyclases sense intracellular as well as extracellular signals. Four original research and 12 review articles sample the extraordinary progress that has been made in the field.

The topic introduces the present status of the field with a figure illustrating that cyclic GMP is generated by two structurally different guanylate cyclases, soluble and the membrane form (Figure 1: Sharma and Duda, 2014). The synthetic machinery and the modes of their operation by which they generate cyclic GMP are entirely different, and so are their physiologically regulated processes. Since many of the membrane guanylate cyclases carry multiple names and all names remain in use, the review also provides a guide to the current nomenclature of the membrane guanylate cyclases (Introductory text Table 1). Then it moves on with a lively historical perspective, replete with unexpected twists and turns. Current research suggests that new developments will continue to follow a tortuous but always exciting path! Guanylate cyclase activating proteins or GCAPs are neuronal calcium sensing proteins that serve as subunits in the ROS-GC complex. The review article by Lim et al. (2014) delves into the molecular mechanism by which GCAPs inhibit GC activity when [Ca^2+^]_i_ is high and stimulate it when [Ca^2+^]_i_ is low. As it turns out, GCAP1 has a more subtle use for its myristoyl tail, compared to other, related neuronal calcium sensors. Besides the additional forms of GCAPs that are being discovered (cf. Wen et al., 2014), ROS-GC1 is subject to modulation by other Ca^2+^ binding proteins: S100B in retinal cones (reviewed by Sharma et al., 2014) and S100B and neurocalcin δ (NCδ) in spermatozoa (Jankowska et al., 2014). Unlike GCAPs, S100B and NCδ stimulate ROS-GC1 at high [Ca^2+^]_i_. Co-expression of S100B and GCAPs with ROS-GC1 in the same cells empowers ROS-GC1 with the ability to operate as a novel, bimodal Ca^2+^ switch wherein guanylate cyclase activity is elevated at very high and at very low [Ca^2+^]_i_. Transduction of Ca^2+^ signals is not restricted to ROS-GCs; NCδ serves as a subunit for the transduction of the ANF signal by ANF-RGC (Duda et al., 2014). Thus, NCδ, in addition to being a “neuronal” calcium sensor, takes on responsibilities outside of neurons. If other guanylate cyclases possess the proper sequences for NCδ, S100B and/or GCAPs interactions, their calcium sensing paradigms will unfold in the future.

Interestingly, GCAP2 has an alternate binding partner that is not a guanylate cyclase. At the photoreceptor synapse, it interacts with RIBEYE to control ribbon size (reviewed by Schmitz, 2014). Recent work on a new ROS-GC binding partner, RD3, (reviewed by Molday et al., 2014) reveals that it is necessary for the intracellular transport of ROS-GC. It also inhibits ROS-GC possibly as a means of suppressing unwanted activity until the cyclase arrives at the proper cellular location.

In retinal rods and cones, cyclic GMP produced by ROS-GCs opens cyclic nucleotide gated cation channels in readiness for visual transduction. Wen et al. (2014) describe the rationale for expressing multiple forms of guanylate cyclases and GCAPs in order to adjust photon response amplitude and quicken photoresponse kinetics according to the requirements of the individual photoreceptor. Besides photoreceptors, there are cyclic GMP pathways in place elsewhere in the retina. Dhingra et al. (2014) have begun to probe the function of these pathways in PDE9A knockout mice by ERG recording.

Downstream signaling pathways for natriuretic peptide receptor GCs are more complex and have not yet been so well characterized (reviewed by Pandey, 2014). In different systems, cyclic GMP synthesis by natriuretic peptide receptor guanylate cyclases decreases cyclic AMP, Ca^2+^, and inositol triphosphate, and downregulates PKC (protein kinase C) and mitogen-activated protein kinases. Barmashenko et al. (2014) present intriguing experiments in rats with reduced expression of C-type natriuretic peptide receptor guanylate cyclase, CNP-RGC. In neuronal recordings from hippocampus, long term potentiation was enhanced while long term depression was reduced. The changes in neuronal excitability were accompanied by increased exploratory behavior and improved object recognition.

Failure to synthesize cyclic GMP properly can cause severe forms of blindness in early childhood. Disruptions in RD3 cause Leber congenital amaurosis (Molday et al., 2014). In a research article, Zägel and Koch (2014) explain how three separate point mutations that cause another form of Leber congenital amaurosis, progressive cone degeneration, and juvenile retinitis pigmentosa, respectively, alter ROS-GC1 biochemistry. Boye (2014) summarizes promising results of experiments in which gene replacement therapy was used to correct faulty ROS-GC1 function in animal models with recessive retinal disease. Prospects for clinical trials are discussed. Dominant forms of retinal degeneration call for a different approach. The application of RNA interference to attenuate faulty ROS-GC activity caused by mutations in GCAP1 suggests that it may be a viable option (reviewed by Jiang et al., 2014). Glaucoma is yet another major cause of blindness, in this case, due to compromise of retinal ganglion cell function. Buys et al. (2014) examine studies on a major risk factor, elevated intraocular pressure, and the link to cyclic GMP synthesis by both soluble and membrane guanylate cyclases. We conclude the topic with a review by Hannig et al. (2014), that outlines the involvement of STa-RGC in the sensation of visceral pain and how a synthetic peptide may provide relief to patients suffering from abdominal pain.

## Conflict of interest statement

The authors declare that the research was conducted in the absence of any commercial or financial relationships that could be construed as a potential conflict of interest.

## References

[B1] Barmashenko G. Buttgereit J. Özcelik C. Herring N. Bader M. Manahan-Vaughan D.. (2014). Regulation of hippocampal synaptic plasticity thresholds and changes in exploratory and learning behavior in dominant negative NPR-B mutant rats. Front. Mol. Neurosci. 7:95. 10.3389/fnmol.2014.00095 25520616 PMC4249455

[B2] Boye S. E. (2014). Insights gained from gene therapy in animal models of retGC1 deficiency. Front. Mol. Neurosci. 7:43. 10.3389/fnmol.2014.00043 24860425 PMC4030156

[B3] Buys E. S. Potter L. R. Pasquale L. R. Ksander B. R. (2014). Regulation of intraocular pressure by soluble and membrane guanylate cyclases and their role in glaucoma. Front. Mol. Neurosci. 7:38. 10.3389/fnmol.2014.00038 24904270 PMC4032937

[B4] Dhingra A. Tummala S. R. Lyubarsky A. Vardi N. (2014). PDE9A is expressed in the inner retina and contributes to the normal shape of the photopic ERG waveform. Front. Mol. Neurosci. 7:60. 10.3389/fnmol.2014.00060 25018695 PMC4073215

[B5] Duda T. Pertzev A. Sharma R. K. (2014). Atrial natriuretic factor receptor guanylate cyclase, ANF-RGC, transduces two independent signals, ANF and Ca^2+^. Front. Mol. Neurosci. 7:17. 10.3389/fnmol.2014.00017 24672425 PMC3955944

[B6] Hannig G. Tchernychev B. Kurtz C. B. Bryant A. P. Currie M. G. Silos-Santiago I. (2014). Guanylate cyclase-C/cGMP: an emerging pathway in the regulation of visceral pain. Front. Mol. Neurosci. 7:31. 10.3389/fnmol.2014.00031 24795564 PMC3997039

[B7] Jankowska A. Sharma R. K. Duda T. (2014). Ca^2+^-modulated ROS-GC1 transduction system in testes and its presence in the spermatogenic cells. Front. Mol. Neurosci. 7:34. 10.3389/fnmol.2014.00034 24808824 PMC4010774

[B8] Jiang L. Frederick J. M. Baehr W. (2014). RNA interference gene therapy in dominant retinitis pigmentosa and cone-rod dystrophy mouse models caused by GCAP1 mutations. Front. Mol. Neurosci. 7:25. 10.3389/fnmol.2014.00025 24778606 PMC3985072

[B9] Lim S. Dizhoor A. Ames J. B. (2014). Structural diversity of neuronal calcium sensor proteins and insights for activation of retinal guanylyl cyclase by GCAP1. Front. Mol. Neurosci. 7:19. 10.3389/fnmol.2014.00019 24672427 PMC3956117

[B10] Molday L. L. Jefferies T. Molday R. S. (2014). Insights into the role of RD3 in guanylate cyclase trafficking, photoreceptor degeneration, and Leber congenital amaurosis. Front. Mol. Neurosci. 7:44. 10.3389/fnmol.2014.00044 24904271 PMC4033307

[B11] Pandey K. N. (2014). Guanylyl cyclase/natriuretic peptides receptor-A signaling antagonizes phosphoinositide hydrolysis, Ca^2+^ release, and activation of protein kinase C. Front. Mol. Neurosci. 7:75. 10.3389/fnmol.2014.00075 25202235 PMC4141235

[B12] Schmitz F. (2014). Presynaptic [Ca^2+^] and GCAPs: aspects on the structure and function of photoreceptor ribbon synapses. Front. Mol. Neurosci. 7:3. 10.3389/fnmol.2014.00003 24567702 PMC3915146

[B13] Sharma R. K. Duda T. (2014). Membrane guanylate cyclase, a multimodal transduction machine: history, present, and future directions. Front. Mol. Neurosci. 7:56. 10.3389/fnmol.2014.00056 25071437 PMC4079103

[B14] Sharma R. K. Makino C. L. Hicks D. Duda T. (2014). ROS-GC interlocked Ca^2+^-sensor S100B protein signaling in cone photoreceptors: review. Front. Mol. Neurosci. 7:21. 10.3389/fnmol.2014.00021 24723847 PMC3972482

[B15] Wen X.-H. Dizhoor A. M. Makino C. L. (2014). Membrane guanylyl cyclase complexes shape the photoresponses of retinal rods and cones. Front. Mol. Neurosci. 7:45. 10.3389/fnmol.2014.00045 24917784 PMC4040495

[B16] Zägel P. Koch K.-W. (2014). Dysfunction of outer segment guanylate cyclase caused by retinal disease related mutations. Front. Mol. Neurosci. 7:4. 10.3389/fnmol.2014.00004 24616660 PMC3935488

